# Effect of Serotonin Reuptake Inhibitors on Pulmonary Hemodynamics in Humans

**DOI:** 10.4021/jocmr654w

**Published:** 2011-09-26

**Authors:** Hilde Pleym, Guri Greiff, Tom Mjorndal, Roar Stenseth, Alexander Wahba, Olav Spigset

**Affiliations:** a Department of Cardiothoracic Anesthesia and Intensive Care, St. Olav University Hospital, Trondheim, Norway; b Department of Circulation and Medical Imaging, Norwegian University of Science and Technology, Trondheim, Norway; c Department of Clinical Pharmacology, Norrland University Hospital, Umea, Sweden; d Department of Cardiothoracic Surgery, St. Olav University Hospital, Trondheim, Norway; e Department of Clinical Pharmacology, St. Olav University Hospital, Trondheim, Norway; f Department of Laboratory Medicine, Children’s and Women’s Health, Norwegian University of Science and Technology, Trondheim, Norway

## Abstract

**Background:**

Serotonin promotes pulmonary arterial vasoconstriction and pulmonary arterial smooth muscle cell proliferation, thereby having the potential to increase pulmonary arterial blood pressure. Although serotonin reuptake inhibitors (SRIs) might inhibit further deterioration in patients with manifest pulmonary arterial hypertension, they may induce pulmonary hypertension in healthy newborns after fetal exposure. As it is unclear whether treatment with SRIs affects pulmonary hemodynamics in adults without pulmonary hypertension, the aim of the present study was to investigate the effect of SRIs on pulmonary hemodynamics in such subjects.

**Methods:**

Sixteen patients with stable angina pectoris scheduled for first time coronary artery bypass grafting were included in the study. Of these 8 were currently treated with an SRI (the SRI group) and 8 were not (the control group). Pulmonary arterial pressures were measured before induction of anesthesia by means of a pulmonary artery catheter. Serotonin transporter and 5-HT_2A_ receptor gene polymorphisms and platelet 5-HT_2A_ receptor expression were studied to elucidate their possible role as modifying factors.

**Results:**

No patients in any of the groups had pulmonary arterial hypertension. Mean pulmonary artery pressure was 15.0 mmHg in the SRI group and 14.5 mmHg in the control group (P = 0.50; 95% confidence interval for the difference, -2.9 to +3.9 mmHg). Neither were there any significant differences between the groups for any of the other hemodynamic variables studied. The various gene polymorphisms and the extent of platelet 5-HT_2A_ receptor expression did not influence the hemodynamic variables.

**Conclusions:**

SRI treatment did not significantly influence pulmonary hemodynamics in patients without pulmonary hypertension.

**Keywords:**

Serotonin; Selective serotonin reuptake inhibitors; Pulmonary hemodynamics; Pulmonary hypertension

## Introduction

Serotonin (5-hydroxytryptamine, 5-HT) promotes both pulmonary arterial vasoconstriction and pulmonary arterial smooth muscle cell proliferation [1]. Elevated levels of circulating peripheral serotonin have been associated with the development of pulmonary arterial hypertension [2,3], with proliferation of pulmonary arterial smooth muscle cells causing increased arterial wall thickness as an important component [4]. The pulmonary arterial smooth muscle cell proliferation is believed to be mediated by uptake of serotonin in these cells via the serotonin transporter. The vasoconstrictive effect of serotonin is mainly thought to be mediated by activation of serotonin (5-HT) receptors on pulmonary arterial smooth muscle cells [5,6], and these receptors may also to some extent contribute to the vascular remodeling process [7].

Serotonin was first linked to pulmonary arterial hypertension in the late 1960s through the association between the use of anorectic agents and the development of pulmonary arterial hypertension. Although somewhat controversial, one suggested mechanism is that anorectic drugs such as aminorex and dexfenfluramine increase circulating serotonin levels, with subsequent serotonin receptor stimulation [3]. Even though they also inhibit the serotonin transporter, decreasing the levels of intracellular serotonin [8], the risk of pulmonary arterial hypertension has during the last two decades been confirmed to be substantially increased during treatment with these drugs [9].

Selective serotonin reuptake inhibitors (SSRIs) such as fluoxetine, citalopram, paroxetine and sertraline are widely used in the treatment of depression and anxiety disorders. In animal models, serotonin-induced proliferation of pulmonary artery smooth muscle cells as well as experimentally induced pulmonary arterial hypertension is inhibited by SSRIs [10-12]. Notably, the mechanism of action of these drugs is to block the function of the serotonin transporter. In humans, SSRI use was in a retrospective cohort study of pulmonary arterial hypertension patients associated with a 50% reduction in the risk of death, although the difference was not statistically significant [13]. Recently, another cohort study [14] showed a significantly reduced mortality (Hazard Ratio 0.35, 95% confidence interval 0.14-0.87) in patients with pulmonary arterial hypertension taking SSRIs.

Although SSRIs might protect against pulmonary arterial hypertension and tend to lower pulmonary arterial blood pressure in adults, the situation seems to be the opposite in newborns after fetal exposure. In two studies, the risk of persistent pulmonary arterial hypertension was increased 6.1-fold and 3.6-fold, respectively, after maternal use of SSRIs in late pregnancy [15,16]. A third study [17] could, on the other hand, not replicate these findings.

The serotonin transporter is encoded by a single gene, SERT (5-HTT, SCL6A4). A polymorphism on the upstream promoter region of the SERT gene has been characterized with two separate forms, long (L) and short (S). The L allele induces an increased rate of gene transcription compared to the S allele, thereby increasing the expression of the transporter. In animal models, overexpression of the human SERT gene has resulted in more severe hypoxia-induced pulmonary arterial hypertension [18]. In a study of 11 children with idiopathic pulmonary hypertension 81% were homozygous for the L allele, as compared to 50% in the control group [19]. In another clinical study, the L allele was found in 65% of patients with pulmonary arterial hypertension, but only in 27% of the control subjects [20]. In a third study, 56% of patients with secondary pulmonary arterial hypertension were homozygous for the L allele, as compared to 27% in the control group [4]. In contrast, two other studies were unable to verify these findings [21,22].

Several subtypes of serotonin receptors, including the 5-HT_1B_, 5-HT_2A_ and 5-HT_2B_ receptors, have been suggested to mediate the vasoconstrictive effect of serotonin. Species differences might exist as to which receptor is the most central. In rats, 5-HT_2A_ receptor blockade inhibits the development of pulmonary arterial hypertension [5]. The importance of this receptor might, however, be lower in humans, as the 5-HT_2A_ receptor antagonist ketanserin is not particularly effective in the treatment of primary pulmonary arterial hypertension [1,23]. Some evidence suggest that the 5-HT_1B_ receptor could possibly be more important for the vasoconstriction in humans [24] and it also might be co-involved with the serotonin transporter in the proliferation of pulmonary arterial smooth muscle [7]. Based upon a single clinical case, it has been suggested that functional loss of 5-HT_2B_ receptor function may increase the risk of pulmonary arterial hypertension [25], but apart from this report there is no clear evidence that the 5-HT_2B_ receptor is involved in vasoconstriction or vascular smooth muscle cell proliferation in humans.

The aim of the present study was to investigate whether serotonin reuptake inhibitors affect pulmonary hemodynamics in patients without pulmonary hypertension, and to elucidate whether various SERT and 5-HT_2A_ receptor gene polymorphisms and the expression of the 5-HT_2A_ receptor could act as modifying factors.

## Methods

The study was a prospective and observational investigation. After approval from the Regional Committee for Medical Research Ethics, Mid Norway, 16 Caucasian patients with stable angina pectoris scheduled for first time coronary artery bypass grafting (CABG) were included after giving written informed consent. The patients were stratified in two groups, one group consisting of 8 patients presently treated with a serotonin reuptake inhibitor (SRI) for more than six weeks (SRI group), and one group consisting of 8 patients not previously or presently treated with an SRI (control group). In the SRI group, 5 patients were treated with citalopram, 2 with escitalopram, and 1 with venlafaxine. A preoperative transthoracic echocardiographic examination of the heart was performed in all patients eligible for participation in the investigation. Patients with any kind of valve disease or other cardiac pathology that could possibly influence pulmonary artery pressures were excluded, as were patients with an ejection fraction (EF) below 0.40. All patients who were asked to participate in the study consented. Enrolled patients from the SRI group and control group were matched in pairs for age and gender, the age matching allowed for an age difference of ± 5 years.

### Measurement of hemodynamic variables

Before surgery, all patients had a radial artery catheter (Becton Dickinson, North Ryde, Australia), and a pulmonary artery catheter (Edwards Lifesciences, Irvine, CA, USA) inserted under local anesthesia with lidocaine 10 mg/mL (Xylocain, Astra Zeneca, Södertälje, Sweden). No analgesics or sedatives were given to the patients before or during catheter insertion. The radial artery catheter and the distal and side ports of the pulmonary artery catheter were connected to pressure transducers (Edwards Lifesciences) and Datex-Ohmeda monitoring software (Helsinki, Finland) for pressure measurements. Correct positioning of the pulmonary artery catheter was confirmed by pressure tracings read from the distal port of the catheter. After completion of the insertion procedure, all patients rested in bed in the supine position for 20 minutes. Thereafter, measurements and calculations of heart rate (HR), mean arterial pressure (MAP), central venous pressure (CVP), systolic pulmonary artery pressure (SPAP), mean pulmonary artery pressure (MPAP), diastolic pulmonary artery pressure (DPAP), pulmonary capillary wedge pressure (PCWP), cardiac output (CO), systemic vascular resistance (SVR), and pulmonary vascular resistance (PVR) were carried out. These variables were all recorded at end-expiration. CO was measured by a thermodilution technique, injecting normal saline of room temperature into the side port of the pulmonary artery catheter. Pulmonary arterial hypertension was defined as MPAP above 25 mmHg [26,27]. When the measurements and calculations were completed, the patients were taken to the operation theater. All patients went through first-time CABG with the use of the left internal thoracic artery and saphenous vein bypass grafts. Cardiopulmonary bypass (CPB) was employed in all cases.

### Drug concentrations in serum

Blood samples for analysis of the SRIs were obtained from the arterial line 12 to 26 hours after intake of the last dose. After centrifugation, serum was separated and the drugs analyzed with liquid chromatography-mass spectrometry (LC-MS) methods published previously [28]. In brief, the analytes were extracted from serum with liquid-liquid extraction, separated on C18 columns and quantitated on an Agilent MSD 1100 LC-MS system (Agilent Technologies, Palo Alto, CA). The limits of quantitation were 10 nmol/L for all analytes. The inter-assay coefficients of variation were less than 10% for venlafaxine and O-desmethylvenlafaxine, and less than 7% for citalopram and escitalopram. The methods were linear in the concentration ranges achieved.

### 5-HT_2A_ receptor binding

The method for radioligand binding to the platelet 5-HT_2A_ receptor has been presented in detail elsewhere [29]. In brief, blood was obtained from the 20 gauge radial artery catheter and collected into polyethylene tubes containing 1.6 mg EDTA (ethylenediaminetetraacetate) per mL blood. Total blood volume obtained was 37.5 mL. Thereafter, platelet-rich plasma was obtained by centrifugation at 180 g for 15 minutes at 20 °C. The platelet pellet was then obtained by centrifugation at 1200 g for 10 minutes at 10 °C and stored frozen until use. On the day of experiment, the platelet pellet was resuspended in hypotonic Tris-buffer, homogenized and centrifuged at 30 000 g for 15 minutes, washed, homogenized and centrifuged once more, and suspended in the incubation buffer. Aliquots of this preparation were incubated for 4 hours at 37 °C in triplicate with seven concentrations of [^3^H]lysergic acid diethylamide ([^3^H]LSD) ranging from 0.25 to 2.5 nM (DuPont N.E.N., Boston, MA, USA). Non-specific binding was assessed in the presence of 300 nM spiperone (Sigma, St. Louis, MO, USA). The binding was terminated by cell harvester filtration through Whatman GF/C (Whatman, Maidstone, Kent, UK) filters prewashed in a 0.3% solution of polyethylenimine. The radioactivity trapped by the filters was determined by liquid scintillation spectroscopy. The final protein concentrations were measured, and the binding characteristics (B_max_ and K_d_) for [^3^H]LSD binding were calculated from Scatchard analysis of the specific binding data according to the method of least squares linear regression.

### Genotyping

Genomic DNA was extracted from whole blood collected in EDTA-treated tubes, using a Qiagene Blood and Cell Culture DNA kit (Quiagen, Hilden, Germany), according to the manufacturer’s guidelines. The polymorphisms were identified as follows:

For the 5HTTLPR polymorphism the upstream region of the serotonin transporter gene was amplified using primers described previously [30], which obtained a 375 bp or 419 bp fragment (14 or 16 repeat alleles). PCR was carried out in a total volume of 30 μL using 1 x PCR buffer, 1.6 mM of MgCl_2_, 2 x of a mixture of dNTPs (0.5 mM of 7-deaza-dGTP, 0.5 mM of dGTP and 1 mM of dATP, dTTP, dCTP (Roche Bioscience, Palo Alto, CA), 0.5 x DMSO (Sigma), 0.3 μM of each primer and 2.5 U of AmpliTaq Gold (Applied Biosystems, Foster City, CA) and 100 ng of genomic DNA. The conditions for the amplification were an initial denaturing step of 95 ºC for 8 min, followed by 33 cycles of 95 ºC for 30 sec, 66 ºC for 30 sec, 72 ºC for 45 sec, and a final elongation step of 72 ºC for 10 min. The PCR products were separated and detected in ethidium bromide 2.5% NuSieve 3:1 Agarose gel (Lonza Bioscience, Basel, Switzerland).

For the 5HTR2A 102 T > C, Thr25Asn, His452Tyr and 516 C > T polymorphisms a real-time PCR was performed to genotype the polymorphism in a Light Cycler 4.0 system (Roche Bioscience) with a set of specifically designed primers and probes. The 5HTR2A 102 T > C and Thr25Asn polymorphisms were carried out in a duplex PCR in a final volume of 10 μL containing 1 x PCR buffer/MgCl_2_, 1 mM of MgCl2, 0.5 μg of purified BSA 100 x (New England Biolabs, Ipswich, MA), 200 μM mix of each nucleotide, 0.25 μM of primer ex1F, 0.5 μM of primer ex1R, 0.20 μM of each probe, 1 U of Taq DNA Polymerase (Roche) and 30 ng of genomic DNA. The conditions for the amplification were an initial denaturating step of 95 ºC for 2 min, 40 cycles of 95 ºC for 2 sec, 58 ºC for 10 sec, 72 ºC for 12 sec, and a melting curve of 0.5 ºC/sec.

The 5HTR2A His452Tyr polymorphism was carried out in a final volume of 10.5 μL containing 1 x PCR buffer/MgCl_2_, 2.5 mM of MgCl_2_, 0.5 μg of purified BSA 100 x (New England Biolabs), 200 μM mix of each nucleotide, 0.2 μM of primer HTR2A-F, 0.5 μM of primer-anchor ILC HTR2A, 0.15 μM of sensor C, 1 U of Taq DNA Polymerase (Roche Bioscience) and 30 ng of DNA. Amplified conditions were an initial denaturating step of 95 ºC for 1 min, 40 cycles of 95 ºC for 5 sec, 58 ºC for 10 sec, 72 ºC for 12 sec, and a melting curve of 1 ºC/sec. The 5HTR2A 516 C > T polymorphism was performed in a final volume of 10.5 μL containing 1 x PCR buffer/MgCl_2_, 0.5 mM of MgCl2, 0.5 μg of purified BSA 100 x (New England Biolabs), 200 μM mix of each nucleotide, 0.25 μM of primer 516-as, 0.5 μM of primer 516-F, 0.15 μM of each probe, 1 U of Taq DNA Polymerase (Roche) and 30 ng of genomic DNA. The conditions for the amplification were an initial denaturation step of 95 ºC for 2 min, 40 cycles of 95 ºC for 1 sec, 57 ºC for 10 sec, 72 ºC for 10 sec, and a melting curve of 1 ºC/sec.

### Sample size calculation and statistics

No previous investigations on possible differences in pulmonary artery pressures between patients treated with SRIs and control patients were identified. The sample size calculations were based on the following assumptions: In a previous study, the standard deviation of MPAP in CABG patients was found to be 3.1 mmHg [31]. We considered a difference in MPAP between the SRI group and the control group of 5 mmHg or more to be of clinical interest. Given these prerequisites and aiming for a power of 0.8 and an alpha of 0.05, 7 patients had to be included in each group.

Data are presented as means (SD) and medians (range) as appropriate. Statistical analyses were carried out using the program package SPSS for Windows, version 15.0 (SPSS Inc., Chicago, IL, USA). Data were compared using Student’s t-test or Wilcoxon-Mann-Whitney U test for scale variables, and Fisher’s exact test for categorical variables. P values < 0.05 were considered statistically significant.

## Results

Eight patients treated with an SRI for more than six weeks and 8 control patients were included in the study. No patients were excluded after inclusion. Patient characteristics, medical data, and data on surgical procedures are presented in Table 1. In the SRI group, the median time of SRI treatment was 2 years, ranging from 2 months to 10 years. Except for the SRI treatment, preoperative medication did not differ between groups. There were no differences between the two groups with respect to previous or current diseases, including chronic lung diseases, the use of vasoactive or other drugs, or smoking habits. No major complications were seen in any of the study patients.

**Table 1:** Patient Characteristics, Medical Data, and Data on Surgical Procedures

****	**SRI group**	**Control group**	**P value**
Age (years)	60.5 (5.1)	61.0 (3.8)	0.83
Body weight (kg)	90.6 (13.4)	84.9 (7.3)	0.31
Gender (males/females)	5/3	5/3	1.00
Previous myocardial infarction (n)	4	3	1.00
Heart failure (n)	1	1	1.00
Systemic hypertension (n)	4	1	0.20
Diabetes (n)	2	3	1.00
Current smoker (n)	1	1	1.00
Ejection fraction (%)	64.1 (9.9)	63.3 (13.4)	0.89
LVEDP (mmHg)	13 (4.0)	20 (6.1)	0.10
Preoperative blood hemoglobin concentration (g/dL)	13.9 (1.1)	14.0 (1.0)	0.90
Preoperative serum creatinine concentration (µmol/L)	82.5 (12.5)	72.4 (8.4)	0.08
NYHA class I/II/III/IV (n)	0/0/8/0	0/1/7/0	1.00
ASA class 3/4 (n)	6/2	8/0	0.47
Surgical time (min)	137 (27)	140 (19)	0.19
CPB time (min)	58 (13)	59 (16)	0.42
XC time (min)	38 (10)	37 (13)	0.48
Number of distal anastomoses	3 (1-4)	4 (2-5)	0.09

Age, weight, ejection fraction, LVEDP, surgical time, CPB time, XC time, preoperative blood hemoglobin concentration, and preoperative serum creatinine concentration are given as mean (SD), Student’s t-test. Number of bypasses is given as median (range), Wilcoxon-Mann-Whitney U test. Gender, NYHA class, ASA class, number of patients with previous myocardial infarction, heart failure, hypertension, and diabetes, and number of patients with a history of smoking within the last two months prior to surgery are given as numbers in each group, Fisher’s exact test. ASA class: American Society of Anesthesiologists preoperative risk classification; CPB: cardiopulmonary bypass; LVEDP: left ventricular end diastolic pressure; NYHA: New York Heart Association; SRI: serotonin reuptake inhibitor; XC: aortic cross clamp. Data on LVEDP were missing for 3 patients in the SSRI group and 4 patients in the control group. For ejection fraction and preoperative blood hemoglobin concentration, data were missing from one patient in the control group.

Data on hemodynamic measurements and calculations, and B_max_ and K_d_ for [^3^H]LSD binding are presented in Table 2. There were no differences in these variables between the groups. The MPAP ranged from 12 to 19 mmHg in the SRI group, and from 10 to 20 mmHg in the control group. None of the patients had pulmonary arterial hypertension. There were no correlations between the SRI dose, SRI serum concentration or duration of treatment and pulmonary artery pressure. All SRI serum concentrations (Table 3) were within the expected interval related to the daily dose [28], indicating good compliance. There were no correlations between B_max_ or K_d_ for [^3^H]LSD binding and pulmonary artery pressure neither in the SRI group, in the control group, nor in the total material (Table 3).

**Table 2:** Hemodynamic Measurements and Calculations, and B_max_ and K_d_ for [^3^H]LSD Binding to Platelet 5-HT_2A_ Receptors

****	**SRI group**	**Control group**	**P value**
SPAP (mmHg)	23.9 (4.3)	24.6 (5.2)	0.76
MPAP (mmHg)	15.0 (2.5)	14.5 (3.7)	0.50
DPAP (mmHg)	10.5 (3.5)	8.9 (3.0)	0.34
PCWP (mmHg)	8.5 (2.8)	8.3 (3.4)	0.88
MAP (mmHg)	90 (10)	100 (14)	0.13
CVP (mmHg)	3.6 (1.7)	4.0 (3.6)	0.79
HR (beats/min)	62 (8)	55 (10)	0.13
CO (L/min)	5.0 (1.0)	5.2 (0.8)	0.70
SVR (dyne·sec/cm5)	1412 (302)	1511 (383)	0.58
PVR (dyne·sec/cm5)	104 (40)	89 (29)	0.42
B_max_ (fmol/mg protein)	37.6 (13.0)	37.0 (7.4)	0.91
K_d_ (nM)	0.55 (0.32)	0.68 (0.25)	0.37

All variables are given as mean (SD), Student’s t-test. SRI: serotonin reuptake inhibitor; HR: heart rate; MAP: mean arterial pressure; CVP: central venous pressure; SPAP: systolic pulmonary artery pressure; MPAP: mean pulmonary artery pressure; DPAP: diastolic pulmonary artery pressure; PCWP: pulmonary capillary wedge pressure; CO: cardiac output; SVR: systemic vascular resistance; PVR: pulmonary vascular resistance.

**Table 3:** Individual Data for the Patients Included, Including Types and Doses of the Serotonin Reuptake Inhibitors, Mean Pulmonary Arterial Pressures, Platelet 5-HT_2A_ Receptor Binding Data, and Serotonin Transporter and 5-HT_2A_ Receptor Genotypes

**Patient number**	**Age, gender**	**Type and dose of SRI (mg/d)**	**Treatment duration with SRI (months)**	**SRI serum concen-tration (nmol/L)**	**MPAP (mmHg)**	**Bmax, 5-HT_2A_ receptor binding (fmol/mg protein)**	**Kd, 5-HT2A receptor binding (nM)**	**SERT S/L genotype**	**5-HTR2A 102 T > C genotype**
SRI group									
1	60, M	ESC, 20	13	107	12	45.8	0.68	LL	CC
2	57, F	ESC, 10	20	68	12	44.6	1.24	LS	CC
3	61, M	CIT, 10	2	56	17	36.1	0.67	SS	TC
4	55, M	CIT, 20	24^1^	171	16	46.8	0.43	LL	CC
5	64, M	CIT, 40	120^1^	170	16	58.1	0.46	LL	CC
6	57, M	CIT, 40	6	391	15	21.7	0.33	LS	TC
7	59, F	CIT, 20	84^1^	124	19	25.8	0.37	LL	TT
8	71, F	VEN, 75	60^1^	340^2^	13	32.2	0.23	LS	TT
Control group									
9	61, M	-	-	-	10	27.5	0.92	LS	TT
10	66, M	-	-	-	12	43.4	0.58	LS	TC
11	61, F	-	-	-	20	43.4	0.49	SS	TT
12	58, M	-	-	-	13	40.9	0.40	SS	TC
13	56, M	-	-	-	19	41.4	0.58	LS	TC
14	60, M	-	-	-	14	40.4	0.76	SS	TT
15	59, F	-	-	-	11	34.8	1.17	LS	TC
16	67, F	-	-	-	17	24.2	0.57	LL	TC

F: female; M: male; ESC: escitalopram; CIT: citalopram; VEN: venlafaxine; MPAP: mean pulmonary arterial pressure; SERT: serotonin transporter gene; S/L: short/long; 5HTR2A: serotonin 2A receptor gene. ¹Approximate value. ²Sum of the concentration of venlafaxine and the active metabolite O-desmethylvenlafaxine.

Individual data on key genotyping results are given in Table 3. For the 5HTR2A Thr24Asn and 516 C > T polymorphisms no variant alleles were found. For the His452Tyr polymorphism, two subjects (both in the control group) were heterozygous and one subject (in the control group) was homozygous for the variant allele. Neither in the SRI group, in the control group nor in the total material there were any significant differences in mean MPAP between groups with the various genotypes or allele variants.

## Discussion

Under some circumstances [15,16], exposure to SRIs may increase pulmonary artery blood pressure and cause pulmonary arterial hypertension, whereas under other circumstances [10-14], SRIs may lower pulmonary arterial blood pressure and protect against the development of pulmonary arterial hypertension. In the present study, measurements of pulmonary artery pressures by means of a pulmonary artery catheter in eight patients treated with SRIs did not reveal any patient with pulmonary arterial hypertension. When compared to the control group, we found no evidence that SRI treatment increases pulmonary arterial blood pressure significantly in subjects without established pulmonary arterial hypertension. It is, however, a weakness that the number of patients included in the present investigation is small. With such a small number of patients included, the risk of type II errors should always be taken into account. The study was powered to reveal an MPAP difference of 5 mmHg between the SRI group and the control group, and minor SRI effects on pulmonary arterial blood pressure can thus not be excluded. The 95% confidence interval for the mean difference in MPAP of 0.5 mmHg between the groups was -2.9 to +3.9 mmHg, i.e. clearly within the difference of 5 mm Hg considered to be of clinical interest and used in the power calculation.

We have only studied two SSRIs, citalopram and escitalopram, in addition to one serotonin and noradrenalin reuptake inhibitor (SNRI), venlafaxine. However, at doses used in clinical practice, the effect on the serotonin transporter is the same for all drugs belonging to the SSRI/SNRI classes. Moreover, with the exception that venlafaxine has some effect on the noradrenaline transporter, the pharmacodynamic profile of the drugs studied is identical. We thus consider that the principal result of the present study would have been the same irrespective of which serotonin reuptake inhibitors we had included.

There were no differences in B_max_ or K_d_ for platelet 5-HT_2A_ receptor binding between the SRI and control groups. Whereas B_max_ expresses the density of receptors on the platelet membrane, K_d_ represents an inverse value of the affinity of the ligand to the receptor. Given previous findings indirectly indicating that activation of the receptor might play a role for the development of pulmonary arterial hypertension [1,5,23], a hypothesis that higher B_max_ values are associated with higher pulmonary arterial pressures can be raised. However, no such relationships could be revealed in the present study, neither in the SRI group, in the control group nor in the combined group. Again, our study is small with a risk of type II errors. In addition, there is an open question whether the expression of 5-HT_2A_ receptors on the pulmonary artery smooth muscle cells is the same as on the platelets. In the present study, platelets were chosen both because they are readily available and because it has been shown that they are a reliable model for other tissues such as brain tissue with regard to 5-HT_2A_ receptor function. The 5-HT_1B_ and 5-HT_2B_ receptors, which could have been at least as relevant to study as the 5-HT_2A_ receptor [7,24,25] are not expressed in platelets. We were thus precluded from studying ligand binding to these receptors with the current technique.

The various 5HTR2A polymorphisms investigated were not related to any variables of pulmonary hemodynamics, neither in the SRI group, in the control group nor in the combined group. However, for most of these polymorphisms the frequency of the variant allele was so low that the chance to find any associations would be relatively remote. On the other hand, also for the 102 T > C polymorphism, which have a higher frequency of the variant allele, no associations were revealed. This result is consistent with the fact that the 5-HT_2A_ receptor may be of relatively low importance for the regulation of pulmonary arterial blood pressure in humans, in contrast to in rodents [1,5,23].

The L allele of the 5-HTTLPR polymorphism of the SERT (5-HTT) gene has been associated with the development of pulmonary arterial hypertension in some studies [4,19,20] but not in all [21,22]. Our study included a total of 16 subjects, which was the same number as in one of the studies in which a significant association was revealed [19]. In contrast to the previous studies, we did not have subjects with manifest pulmonary arterial hypertension included. Thus, our study can neither confirm nor disprove whether the L allele predisposes to pulmonary arterial hypertension, but there was at least no evidence that the L allele (or the S allele) has any major modifying impact on the possible effect of SRI treatment on pulmonary hemodynamics. The fact that patients in the SRI group were treated for a psychiatric condition should not have introduced bias in our material, as it has been shown that the frequencies of the various L/S genotypes as well as the various genotypes of the 5HTR2A polymorphisms are the same in patients with depression-related disorders as in healthy controls [32].

The present study has a number of weaknesses that should be addressed. As already discussed a major weakness is the low number of patients included, which means that we cannot draw any conclusion regarding any potential mechanistic relationship. Moreover, it makes it unlikely that any genetic effect should be revealed. However, we nevertheless consider genetic testing as being of interest, both in order to further elucidate the complex interrelationship between drug treatment and clinical effects, and to increase the potential to explain unexpected or remarkable findings [33]. Another weakness is that we have not made a thorough genetic investigation of all serotonin genes possibly involved in the regulation of pulmonary hemodynamics. With a larger number of subjects included and also other genetic methods available, haplotype analyses or even microarray methodology could have been employed, thus increasing the possibility of revealing significant associations. Finally, the patients included were not healthy, but highly selected on the basis of their cardiovascular disease. On the other hand, it would not be ethically justified to perform an invasive study like the present in subjects not undergoing cardiac surgery.

We consider the direct measurements of pulmonary arterial pressures by means of a pulmonary artery catheter as being a strength due to the reliability of the method. To our knowledge, no previous investigations have reported direct invasive measurements of pulmonary artery pressures in patients treated with SRIs. Although regarded as a golden standard, the pulmonary artery catheter technique is time consuming and does confer a certain risk to the patient. Therefore, to be able to include a larger number of patients, in the future pulmonary pressures could preferably be measured by less invasive methods such as echocardiography.

### Conclusion

The present study was unable to find any effect of SRI treatment on pulmonary hemodynamics. However, as minor effects cannot be completely excluded, further investigations in larger patient materials, including subjects not undergoing CABG, should be performed before any final conclusions can be drawn. Studies in a larger number of patients would also have the advantage of better utilizing the potential of genetic testing to elucidate possible gene interactions with a possible effect of SRIs on pulmonary hemodynamics.
